# Twiner: correlation-based regularization for identifying common cancer gene signatures

**DOI:** 10.1186/s12859-019-2937-8

**Published:** 2019-06-25

**Authors:** Marta B. Lopes, Sandra Casimiro, Susana Vinga

**Affiliations:** 1 Instituto de Telecomunicações, Instituto Superior Técnico, Universidade de Lisboa, Av. Rovisco Pais 1, Lisboa, 1049-001 Portugal; 2 INESC-ID, Instituto Superior Técnico, Universidade de Lisboa, Rua Alves Redol 9, Lisboa, 1000-029 Portugal; 3 Luis Costa Lab, Instituto de Medicina Molecular, Faculdade de Medicina da Universidade de Lisboa, Avenida Professor Egas Moniz, Lisboa, 1649-028 Portugal; 4 IDMEC, Instituto Superior Técnico, Universidade de Lisboa, Av. Rovisco Pais 1, Lisboa, 1049-001 Portugal

**Keywords:** Gene network, Sparse logistic regression, Breast invasive carcinoma, Triple-negative breast cancer, Prostate adenocarcinoma

## Abstract

**Background:**

Breast and prostate cancers are typical examples of hormone-dependent cancers, showing remarkable similarities at the hormone-related signaling pathways level, and exhibiting a high tropism to bone. While the identification of genes playing a specific role in each cancer type brings invaluable insights for gene therapy research by targeting disease-specific cell functions not accounted so far, identifying a common gene signature to breast and prostate cancers could unravel new targets to tackle shared hormone-dependent disease features, like bone relapse. This would potentially allow the development of new targeted therapies directed to genes regulating both cancer types, with a consequent positive impact in cancer management and health economics.

**Results:**

We address the challenge of extracting gene signatures from transcriptomic data of prostate adenocarcinoma (PRAD) and breast invasive carcinoma (BRCA) samples, particularly estrogen positive (ER+), and androgen positive (AR+) triple-negative breast cancer (TNBC), using sparse logistic regression. The introduction of gene network information based on the distances between BRCA and PRAD correlation matrices is investigated, through the proposed *twin networks recovery* (twiner) penalty, as a strategy to ensure similarly correlated gene features in two diseases to be less penalized during the feature selection procedure.

**Conclusions:**

Our analysis led to the identification of genes that show a similar correlation pattern in BRCA and PRAD transcriptomic data, and are selected as key players in the classification of breast and prostate samples into ER+ BRCA/AR+ TNBC/PRAD tumor and normal tissues, and also associated with survival time distributions. The results obtained are supported by the literature and are expected to unveil the similarities between the diseases, disclose common disease biomarkers, and help in the definition of new strategies for more effective therapies.

## Background

Breast invasive carcinoma (BRCA) and prostate adenocarcinoma (PRAD) are the two most common invasive cancers in women and men, respectively [1]. In both types of cancers, the vast majority of cases are hormone-dependent, meaning that tumor growth is deeply related to hormone-related signaling pathways. About 70% of all BRCA are estrogen receptor (ER) and/or progesterone receptor (PR) positive (ER+ and/or PR+), and endocrine treatment can be effective in all stages of disease [2].

In the PRAD case, androgen/androgen receptor (AR) signaling pathway is deeply involved in the progression of the disease, and androgen deprivation therapy (ADT) with anti-androgens remains as the main treatment in early and late stage disease [3]. Hormone-dependent signaling pathways like ER, PR or AR, ultimately regulate numerous cell functions, and positively impact cell proliferation [4, 5]. However, ER and AR signaling are not exclusively important in BRCA and PRAD, respectively. It is known that estrogens play an important role in male sex hormone secretion, in the physiology of normal prostate tissues, and in prostate carcinogenesis. In fact, selective targeting of ER *α* or *β* may be an option in the treatment of castration resistant metastatic prostate cancer cells [6]. By the other hand, AR is expressed in about 80% of primary breast cancers, particularly in triple-negative breast cancer (TNBC), characterized by lack of expression of estrogen receptor 1 (*ER*), progesterone receptor (*PR*), and human epidermal growth factor receptor type 2 (*HER2*) [7], and associated with a poor prognosis [8]. AR-inhibiting drugs have indeed shown antitumorigenic activity in preclinical and proof-of-concept clinical studies in TNBC [9].

Given that BRCA and PRAD are hormone-dependent cancers, possibly sharing layers of signaling and regulatory pathways, it is important to unravel common players that could establish a link between the hormone-dependence and the fact that both types of cancers exhibit a high tropism to bone. To achieve that, one possible solution is to, given a classification model, extract the most relevant features in the discrimination between tumor and normal tissue, out of the full set of dozens of thousands of features currently delivered by high-throughtput ‘omic technologies. Classification of tumor and normal tissue can be performed separately for BRCA and PRAD, however, given the similarities between the two diseases, a common strategy to classify patients tissue, while simultaneously identifying the genes playing a role in both diseases, would be of great value. This way, a considerable reduction in the effort in defining new therapies could be accomplished.

Statistical learning in high-dimensional ‘omic data poses many challenges, in particular for parameter estimation, since the models are seldom identifiable. One way to cope with this problem is to add constraints in the parameters space. For instance, imposing sparsity in the solution will enable feature selection since a large subset of the parameters will be exactly zero. Several methods have been applied in the context of ‘omic data, namely the lasso and elastic net, which impose a *l*_1_ regularizer and a linear combination of *l*_1_ (lasso) and *l*_2_ (ridge) penalties, respectively [10, 11]. While the ridge penalty cannot shrink coefficients exactly to zero, therefore keeping all the variables in the model, the lasso estimator enables performing variable shrinkage and selection at the same time, making the solution sparse.

Model constraints can benefit from external knowledge on the biological disease processes, often given by network information. For example, groups of genes are co-expressed under certain conditions or their protein products interact with each other to carry out a biological function [12], which can be represented by graphs. Given a graph *G*:=(*V*,*E*), *V* denotes vertices (or nodes) and *E* the set of edges. In a gene network, vertices are genes and edges represent a weighted relation between two genes. It has been advocated that incorporating network information as a constraint in the loss function potentially increases the model predictive performance of, e.g., sparse Cox and logistic regression models, as shown when modeling the survival of ovarian cancer carcinoma patients and classifying patients into breast cancer subtypes [12–14]). Furthermore, including network-based regularizers may improve model interpretability since prior knowledge/information via constraints will drive parameter estimation towards meaningful biological solutions. Such network information can be either obtained by a priori defined pathways and network interactions available in public databases, by de novo construction of specific subnetworks from the set of mutated or differentially expressed genes (e.g., [15, 16]), or by the data correlation itself [13].

In this work we combine correlation-based regularizers and sparse logistic regression to solve a binary classification problem with BRCA and PRAD cancer tissues, and normal tissue from breast and prostate cancer patients as classes. Different datasets will be considered in two case studies in the search for shared gene signatures in BRCA and PRAD: I) ER+ BRCA vs. PRAD, showing similarities at the ER signaling level and shared marked bone osteotropism; and II) AR+ TNBC vs. PRAD, sharing AR-signaling dependency. With the goal of identifying a common network to each BRCA subtype and PRAD data, ER+ BRCA, AR+ TNBC and PRAD gene correlation networks will be generated using the Pearson correlation between observed (gene expression) variables as similarity measure. For a given gene in the network, the more similar its correlation pattern between the two diseases under consideration is, the less penalized it will be in the regularization term of sparse logistic regression. Selected similarly correlated genes in the two diseases, i.e., playing a role in discriminating between cancer and non-cancer, can be seen as potential biomarkers candidates in the two groups of patients.

## Methods

### Datasets

The transcriptomic data on breast and prostate cancer patients used in this work were obtained from The Cancer Genome Atlas (TCGA) Data Portal (https://cancergenome.nih.gov/).

#### Breast invasive carcinoma (BRCA)

The BRCA RNA-Seq Fragments Per Kilo base per Million (FPKM) dataset was imported using the ‘brca.data’ R package^1^. The BRCA gene expression data is composed of 57251 variables for a total of 1222 samples from 1097 individuals. From those samples, 1102 correspond to primary solid tumor, 7 to metastases and 113 to normal breast tissue. Only samples from primary solid tumor were selected for analysis, from those only ER+ BRCA samples were considered to avoid the introduction of confounding effects by accounting for non-hormonal BRCA tumor information in the search for a common gene signature for BRCA and PRAD. Information on the samples ‘positive’ clinical status for ER was obtained from the BRCA clinical data also available from the TCGA. The BRCA response variable **Y** is binary, coded with ‘1’ for *tumor* (802 samples) and ‘0’ for *normal* (79 samples) tissue.

#### Triple-negative breast cancer (TNBC)

The TNBC dataset was built from the BRCA dataset described above. The TNBC binary response vector **Y** was created, with ‘1’ corresponding to TNBC individuals (with *ESR1*, *PGR* and *ERBB2* ‘negative’ expression), and non-TNBC (‘0’) to non-TNBC (other types of BRCA) patients, whenever at least one of the three genes is ‘positive’. The individuals’ *status* regarding ER, PR and HER2, needed for building **Y**, were obtained from the BRCA clinical data available from the TCGA, composed of 114 variables, as described in Lopes et al. (2018) [17, 18]. Only AR+ TNBC samples (with AR expression larger than the median AR expression over all TNBC samples) were considered for building the TNBC dataset, accounting for 80 *tumor* and 113 *normal* tissue samples.

#### Prostate adenocarcinoma (PRAD)

The PRAD RNA-Seq Fragments Per Kilo base per Million (FPKM) dataset was imported using the ‘prad.data’ R package^2^. The PRAD gene expression data is composed of 57035 variables for a total of 551 samples from 500 individuals. From those samples, 495 correspond to primary solid tumors, 1 to metastases and 52 to normal tissue. Only samples from primary solid tumor were considered for analysis. The PRAD response variable **Y** is binary, coded with ‘1’ for *tumor* samples and ‘0’ for *normal* tissue samples. The PRAD dataset is composed of 495 *tumor* and 52 *normal* samples.

#### Data pre-processing

FPKM normalized ER+ BRCA, AR+ TNBC, and PRAD gene expression data were log-transformed and Z-score normalized prior to data analysis. A subset of ∼ 20000 variables in each dataset was considered for further analysis, corresponding to the protein coding genes reported from the Ensembl genome browser [19] and the Consensus CDS project [20], and shared by each pair of diseases under evaluation (ER+ BRCA vs. PRAD and AR+ TNBC vs. PRAD).

### Classification modeling

#### Sparse logistic regression

Binary logistic regression describes the relationship between one or more independent variables and a binary outcome vector **Y**={*Y*_*i*_}_*i*=1,...,*n*_, which is given by the logistic function

1 $$ P ({Y}_{i}=1|\mathbf{X}_{i})=\frac{\exp\left(\mathbf{X}_{i}^{T} \boldsymbol{\beta}\right)}{1+\exp\left(\mathbf{X}_{i}^{T} \boldsymbol{\beta}\right)},  $$

where **X**_*i*_,*i*=1,...,*n*, is the vector of *p* covariates for observation *i*, *P*(*Y*_*i*_=1|**X**_*i*_) is the probability of success for observation *i*, and ***β***=(*β*_1_,*β*_2_,…*β*_*p*_) are the regression coefficients associated with the *p* independent variables.

The parameters of the logistic model are estimated by maximizing the log-likelihood function of the logistic model, given by

2 $$  l(\boldsymbol{\beta}) \,=\, \!\sum_{i=1}^{n}\! \left\{y_{i} \log P (Y_{i}=1|\!\mathbf{X}_{i}) \!+ \!(1 - y_{i})\! \log\! [1 - P (Y_{i}=1|\!\mathbf{X}_{i})]\!\right\},  $$

where the binary variable *y*_*i*_ indicates success (*y*_*i*_=1) or unsuccess (*y*_*i*_=0) for observation *i*. By the introduction of a regularization term, the log-likelihood function becomes

3 $$ \l\!(\boldsymbol{\beta}) \!= \sum_{i=1}^{n} \!\left\{y_{i}\! \log P (Y_{i}\,=\,1|\mathbf{X}_{i})\! + \!(1 - y_{i})\! \log [1\! -\! P (Y_{i}=1|\!\mathbf{X}_{i})]\!\right\}\! +\! F(\boldsymbol{\beta}),  $$

where

4 $$ F(\boldsymbol{\beta}) = \lambda \left\{\alpha \Vert \boldsymbol{\beta} \Vert_{1} + (1-\alpha) \Vert \boldsymbol{\beta} \Vert^{2}_{2} \right\}  $$

stands for the elastic net penalty, with *α*=1 corresponding to lasso and *α*=0 to ridge, and the tuning parameter *λ* controlling the amount of shrinkage in the coefficients.

#### Correlation-based network regularization

With the specific goal of weighting variables based on their similarities across two given diseases, we propose twiner, a structured regularizer based on the pairwise correlations between variables, independently obtained from two given datasets.

Consider two correlation matrices for diseases *A* and *B*, $\Sigma _{A} = \left [\boldsymbol {\sigma }_{1}^{A},...,\boldsymbol {\sigma }_{p}^{A}\right ]$ and $\Sigma _{B} = \left [\boldsymbol {\sigma }_{1}^{B},...,\boldsymbol {\sigma }_{p}^{B}\right ]$, respectively, where each column $\boldsymbol {\sigma }_{j} \in \mathbb {R}^{p}$ represents the correlation of each gene *j*=1,…,*p* with the remaining ones. The proposed dissimilarity measure *d*_*j*_(*A*,*B*) of gene *j* between *A* and *B* is given by the angle of the corresponding vectors, i.e.,

5 $$ d_{j}(A,B) = \arccos{\frac{<{\boldsymbol{\sigma}}_{j}^{A},{\boldsymbol{\sigma}}_{j}^{B}>}{\Vert {\boldsymbol{\sigma}}_{j}^{A} \Vert \cdot \Vert {\boldsymbol{\sigma}}_{j}^{B} \Vert}}, \quad j=1,\ldots, p.   $$

The rationale of using the angle is that two patterns will be identified as similar if they have the same proportionality between the entries across the two datasets, irrespective of the magnitude of the vectors. In the context of the present application, one gene has a similar role in BRCA and PRAD, if it is similarly correlated with the remaining genes in the two diseases. This correlation-based regularization constitutes the basis of twiner, since this dissimilarity will then be used as a penalization for the cost function. The weighting vector to be used as a penalization **w**=(*w*_1_,...,*w*_*j*_,...,*w*_*p*_) is therefore based on this distance, normalized by their maximum value:

6 $$ w_{j} = \frac{d_{j}(A,B)}{\max_{k} d_{k}(A,B)}, \quad j, k =1,\ldots,p.   $$

The penalty term in Eq. 4 takes the form

7 $$ F(\boldsymbol{\beta}) = \lambda \left\{\alpha \Vert \mathbf{w} \circ \boldsymbol{\beta} \Vert_{1} + (1-\alpha) \Vert \mathbf{w} \circ \boldsymbol{\beta} \Vert^{2}_{2} \right\},  $$

with vector **w** representing the factors that control how much of the penalty *λ* affects each coefficient, and ∘ standing for the element wise (or Hadamard) product.

For a given gene *j*, the smaller the distance between ${\boldsymbol {\sigma }}_{j}^{A}$ and ${\boldsymbol {\sigma }}_{j}^{B}$, the more similar the diseases are regarding the overall gene *j* correlation pattern. Therefore, the resulting *twin networks recovery* (twiner) penalty enables the identification of variables with similar (*twin*) correlation with the remaining variables across the two diseases, with smaller penalties being associated to genes with smaller distances between the diseases’ correlation matrices. The influence these genes have in the outcome will be assessed by regularized logistic regression based on the elastic net penalty.

The modeling strategy described above will be applied in two case studies with the goal of finding common gene signatures in i) ER+ BRCA and PRAD, and ii) AR+ TNBC and PRAD, as described next.

#### Classification of BRCA and PRAD RNA-Seq data

**Fig. 1:**
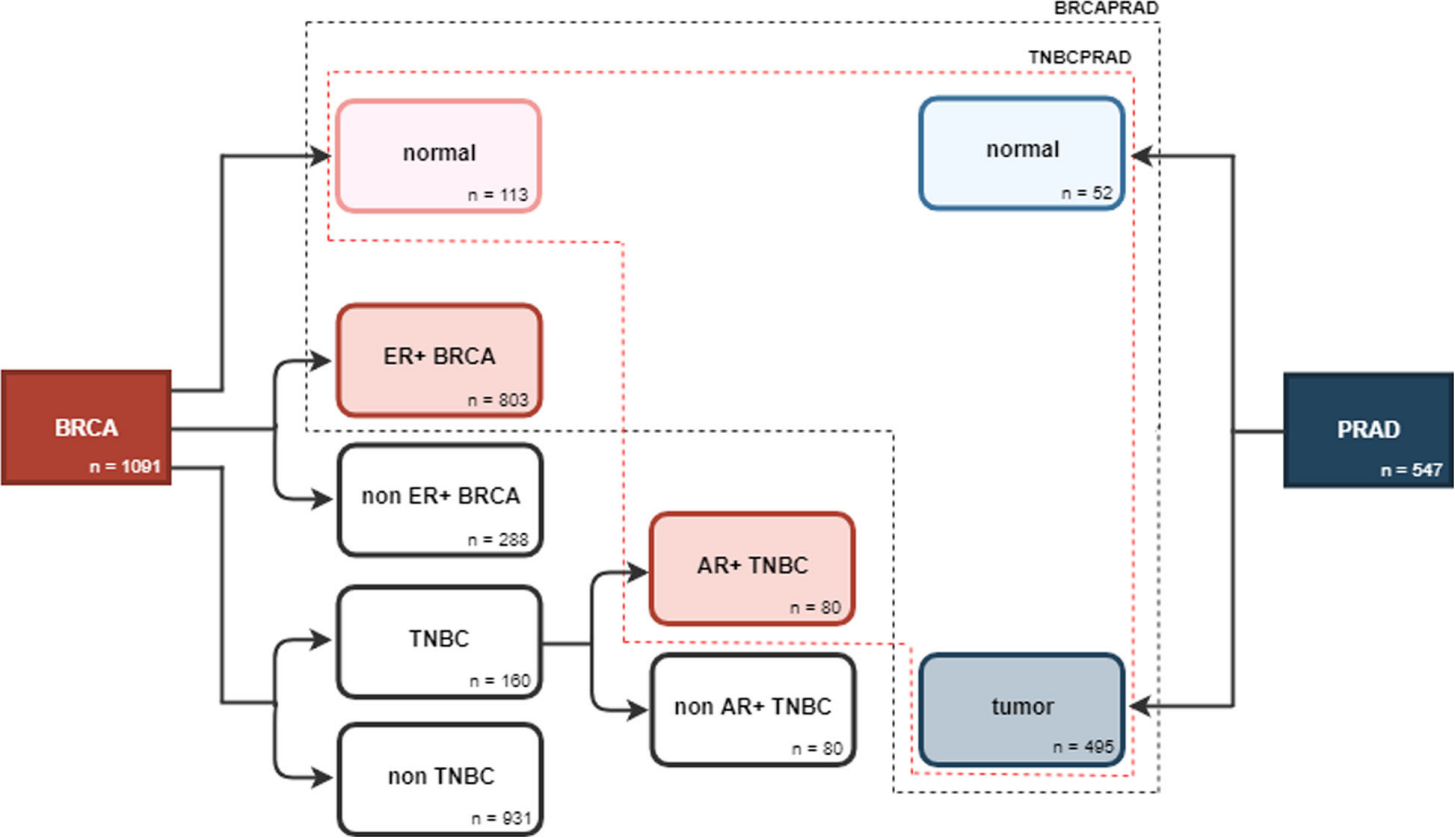
Schematic representation of the procedure to construct the BRCAPRAD (blue dashed line) and TNBCPRAD (red dashed line) datasets analysed. BRCA, Breast Invasive Carcinoma; TNBC, Triple-Negative Breast Cancer; ER+ BRCA, estrogen receptor positive BRCA; AR+ TNBC, androgen receptor positive TNBC; PRAD, Prostate Adenocarcinoma

Sparse logistic regression using the elastic net penalty (EN) was used to classify RNA-Seq data from patients into ER+ BRCA, AR+ TNBC and PRAD vs. *normal* breast and prostate tissue samples. The overall procedure for dataset construction is illustrated in Fig. 1.

With the goal of finding a common gene signature between ER+ BRCA and PRAD cancer types, ER+ BRCA and PRAD data were grouped into a single class, i.e., *tumor*, and EN applied to classify RNA-Seq data into ER+ BRCA or PRAD (*tumor*) vs. *normal* samples, herein called BRCAPRAD model. Three quarters of randomly selected samples were assigned to training samples for model construction, whereas the remaining samples were assigned to test samples for model evaluation. The classification was performed using two models: 1) EN; and 2) sparse logistic regression using the twiner penalty (twiner). For both EN and twiner models the alpha parameter was set to *α*=0.9, which yields a adequate number of features to be further analysed without compromising clinical interpretability. The Pearson correlation matrices from ER+ BRCA and PRAD RNA-Seq data are matrices *Σ*_*A*_ and *Σ*_*B*_, as seen above, and the response **Y** vector is a binary vector with ‘0’ corresponding to *normal* tissue and ‘1’ to *tumor* (ER+ BRCA or PRAD) tissue. The angular distance between ${\boldsymbol {\sigma }}_{j}^{A}$ and ${\boldsymbol {\sigma }}_{j}^{B}$, corresponding to the correlation pattern of variable *j* in matrices *Σ*_*A*_ and *Σ*_*B*_ was used for building the weight vector **w** as explained above. With the goal of searching for shared disease biomarkers, genes showing larger angular distances between correlation vectors in the two diseases were discarded, only keeping those (out of the previous ∼ 20000) showing an angular distance less than 75 ^∘^, at the same time contributing to reduce model complexity. The new dimension in the variables space was thus decreased to 16367 genes.

EN and twiner models were generated 100 times for randomly chosen training and test sets. The median values for the mean squared error (MSE) of classification, the area under the Precision-Recall curve (AUC) [21, 22] and the number of misclassifications, along with the set of variables selected in more than 75% of the runs, were taken for comparison of the modeling strategies employed.

The same analysis was performed in the search for a common gene signature between AR+ TNBC and PRAD cancer types. As for the ER+ BRCA vs. PRAD case, only genes (out of the previous ∼ 20000) showing lower angular distances were considered, yielding 14598 genes for model building in the AR+ TNBC vs. PRAD case. EN and twiner models were generated to classify RNA-Seq data into AR+ TNBC or PRAD *tumor* samples (‘1’) vs. TNBC and PRAD *normal* samples (‘0’), herein called the TNBCPRAD model.

For biological interpretation of the variables selected by the methods, gene correlation networks were represented only for ER+ BRCA, AR+ TNBC, and PRAD data, using the variables exclusively selected by EN, twiner and shared by the two models.

Finally, as an attempt to clinically validate our approach, the variables selected by EN and twiner were tested in a survival analysis on ER+ BRCA, AR+ TNBC and PRAD tumor data, using the Cox regression model [23]. The individuals in each dataset were separated in two groups by the median of the fitted relative risk. This allows to perform the Log-Rank test via the Kaplan-Meier estimator [24], and to assess if the survival curves of the two groups are statistically different by calculating the *p*-values. Increased risk groups’ separability by a given set of genes is expected to trigger further research on the role of these genes in the disease.

Individual models for predicting the class, *tumor* vs. *normal*, independently for ER+ BRCA, AR+ TNBC and PRAD data, herein called ER+ BRCA, AR+ TNBC and PRAD models, were also built, using the same set of variables used for the combined BRCAPRAD and TNBCPRAD models, as explained above. The goal was to identify (if any) genes selected in common by independent disease models (potential shared disease biomarkers), and the overlap with BRCAPRAD and TNBCPRAD sparse logistic models aiming at extracting common gene signatures from two diseases through the twiner penalty. Similarly to the independent BRCAPRAD nd TNBCPRAD models, training and test sets were randomly generated. The optimization of the parameters *λ* and *α* based on the MSE for the models described above was performed by 10-fold cross-validation (CV), with varying *α* values (1 >*α*> 0) tested.

The glmnet R package [25] implemented in the free R statistical software [26] was used in our study for building the above sparse logistic regression models with elastic net regularization. The **w** vector was introduced as penalty factor in the glmnet function. Differentially expressed genes across *tumor* and *normal* ER+ BRCA, AR+ TNBC and PRAD tissue were identified using the limma Bioconductor R package [27], in order to support further clinical analysis and interpretation of the obtained genes.

## Results

### Principal component analysis

**Fig. 2:**
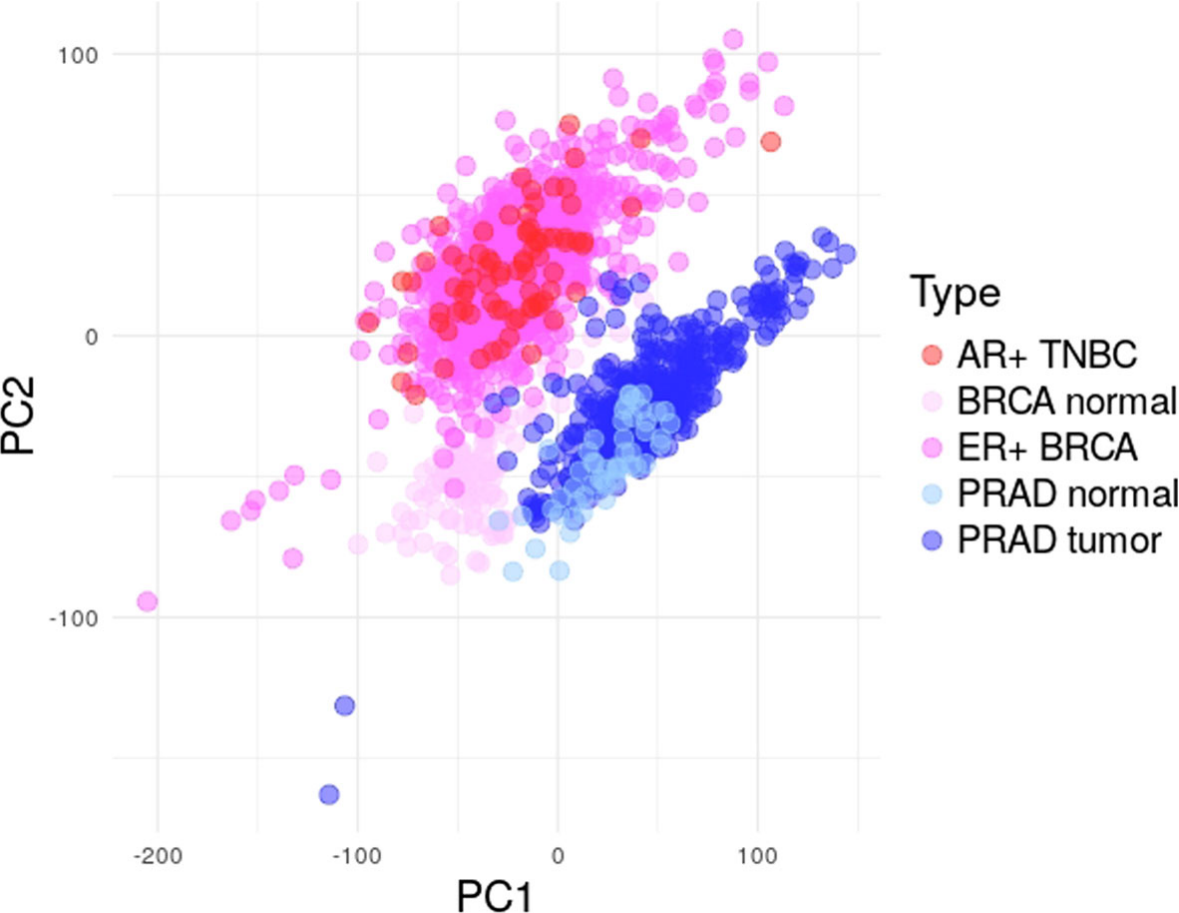
Representation of ER+ BRCA, AR+ TNBC and PRAD tumor and normal samples in the space of the first two principal components. ER+ BRCA, estrogen receptor positive Breast Invasive Carcinoma; AR+ TNBC, androgen receptor positive Triple-Negative Breast Cancer; PRAD, Prostate Adenocarcinoma

Before classification of samples into ER+ BRCA/AR+ PRAD and PRAD *tumor* and *normal* breast and prostate samples, a first non-supervised analysis was intended to visualize samples’ grouping in a reduced dimensional space. A Principal Component Analysis (PCA) was applied to a dataset comprising gene expression data from ER+ BRCA, AR+ TNBC and PRAD *tumor* tissue samples, along *normal* tissue samples from breast and prostate patients. A clear separation between ER+ BRCA/AR+ TNBC and PRAD *tumor* samples is observed in the space of the first two principal components (Fig. 2), though partial overlap is observed when looking at PCs individually. Overlap between ER+ BRCA/AR+ TNBC *tumor* and *normal* samples is absent in PC2, as opposed to that observed for PRAD *tumor* and *normal* samples, showing overlap in both PCs. Finally, great overlap between BRCA (ER+ BRCA) and TNBC (AR+ TNBC) is observed in the subspace represented (Fig. 2).

### Sparse logistic regression

**Fig. 3:**
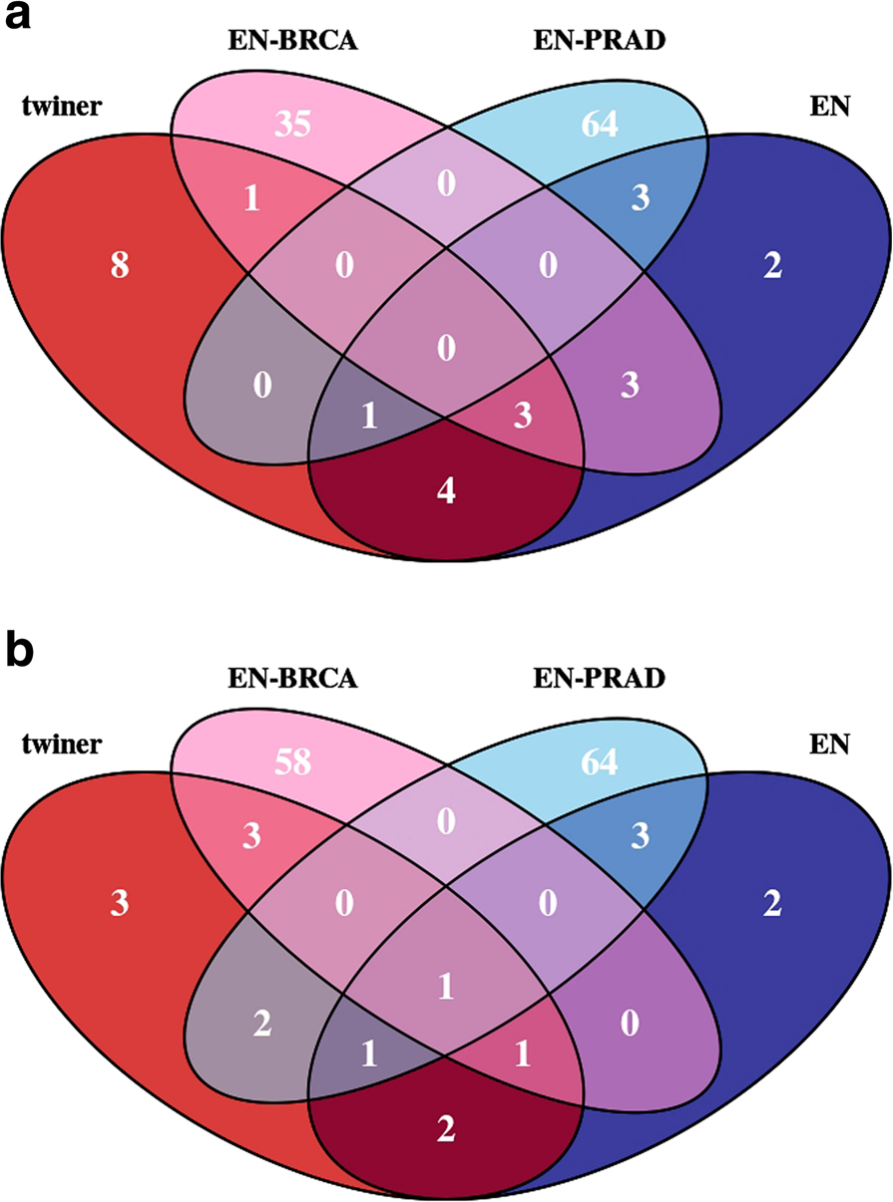
Venn diagrams representing the number of variables selected by elastic net (EN) (blue) and twiner (red), and by EN-BRCA (pink) and EN-PRAD (light blue) individual models, for the two case studies evaluated: **a**) ER+ BRCA vs. PRAD; and **b**) AR+ TNBC vs. PRAD. ER+ BRCA, estrogen receptor positive Breast Invasive Carcinoma; AR+ TNBC, androgen receptor positive Triple-Negative Breast Cancer; PRAD, Prostate Adenocarcinoma

Sparse logistic regression models were built independently for ER+ BRCA, AR+ TNBC and PRAD data for the classification of patients into *tumor* or *normal* tissue. The ER+ BRCA model was based on *α*=0.9, ending up in the selection of 42 variables and 1 misclassification in the test set (Table 1). The model generated for AR+ TNBC dataset was based on an *α* value of 0.8, yielding the selection of 63 variables and no misclassifications in both training and test sets. The PRAD model selected 68 variables, considering an optimum *α* value of 0.5, and misclassified 11 patients in the training set and 6 in the test set. A higher number of misclassifications was obtained for the PRAD dataset, as foreseen by PCA (Fig. 2). No variables were selected in common between the ER+ BRCA and PRAD models (Fig. 3a), whereas only one variable, gene *NKAPL*, was selected in common between the TNBC and PRAD models using this strategy (Fig. 3b).

**Table 1:** Summary of BRCA, TNBC and PRAD EN models (MSE, mean squared error; AUC, area under the precision-recall curve; Miscl, misclassifications; Vars, nr. of variables selected)

	*α*	# Vars	MSE		AUC		# Miscl	
			Train	Test	Train	Test	Train	Test
BRCA	0.9	42	0.0001	0.0065	1	1	0	1
TNBC	0.8	63	0.0002	0.0025	1	1	0	0
PRAD	0.5	68	0.0187	0.0309	0.97	0.97	11	6

#### ER+ BRCA vs. PRAD

EN and twiner were applied to the BRCAPRAD dataset, as a means to identify a common gene signature between ER+ BRCA and PRAD diseases. Summary results of the two modeling strategies applied to 100 random training and test sets can be found in Table 2. A median MSE decrease in 11% and 4% for the training and test sets was observed, respectively (Table 2). Three genes were always selected by the 100 EN models (*BGN*, *GLRA4* and *NKAPL*) and 2 by twiner models (*NKAPL* and *PAK3*), with *NKAPL* being selected in common by the two modelling strategies.

**Table 2:** Summary of BRCAPRAD and TNBCPRAD model results by EN and twiner, considering the median values obtained from 100 models built on randomly generated training and test sets (MSE, mean squared error; AUC, area under the precision-recall curve; Miscl, misclassifications; Vars, nr. of variables selected)

		# Vars	MSE		AUC		# Miscl	
			Train	Test	Train	Test	Train	Test
BRCAPRAD	EN	58	0.013	0.018	0.98	0.98	17	9
	twiner	69	0.012	0.018	0.99	0.98	15	9
TNBCPRAD	EN	61	0.025	0.034	0.97	0.96	16	8
	twiner	71	0.025	0.034	0.97	0.96	14	8

**Fig. 4:**
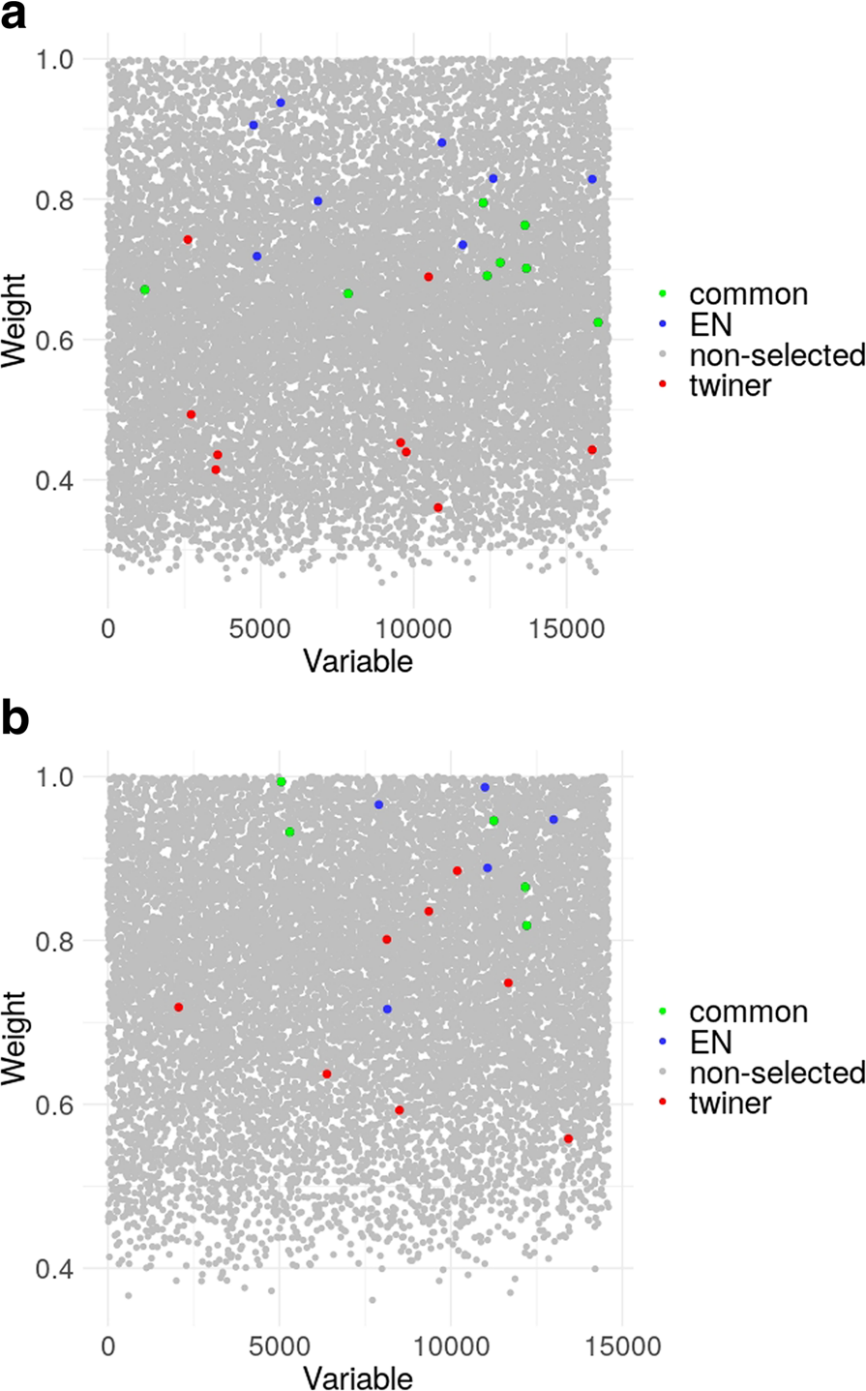
Weights of the variables selected by elatic net (EN) (blue) and twiner (red), and selected in common by the two modeling strategies (green), for the two case studies evaluated: **a**) ER+ BRCA vs. PRAD; and **b**) AR+ TNBC vs. PRAD. ER+ BRCA, estrogen receptor positive Breast Invasive Carcinoma; AR+ TNBC, androgen receptor positive Triple-Negative Breast Cancer; PRAD, Prostate Adenocarcinoma

Seventeen genes were selected in more than 75 out of 100 twiner models (Fig. 3; Table 3), 8 in common with EN (*CXCR2*, *GLRA4*, *LRRC3B*, *NKAPL*, *PAK3*, *RP11-729L2.2*, *SCN5A* and *TMEM236*) and the remaining 9 (*BMT2*, *CLEC11A*, *CSGALNACT2*, *HMGCS1*, *POLR2H*, *RP11-371E8.4*, *SCARA5*, *SLC17A7* and *ZBTB24*) exclusively selected by twiner (Fig. 3; Table 3). Out of the genes selected by twiner, 4 (*GLRA4*, *LRRC3B*, *PAK3* and *SLC17A7*) were shared with the BRCA model and 1 (*NKAPL*) with the PRAD model, respectively (Table 3). Most genes from the 9 genes exclusively selected by twiner (Fig. 3a) have lower weights compared to those exclusively selected by EN and selected in common by the two strategies (Fig. 4a), meaning that their correlation pattern across the genes space is more similar between ER+ BRCA and PRAD cancer types, compared to the remaining genes selected.

**Table 3:** Genes selected by EN and twiner; pink and blue arrows indicate up- (*↑*) and down-regulated (*↓*) genes in ER+ BRCA, AR+ TNBC and PRAD, respectively

**Fig. 5:**
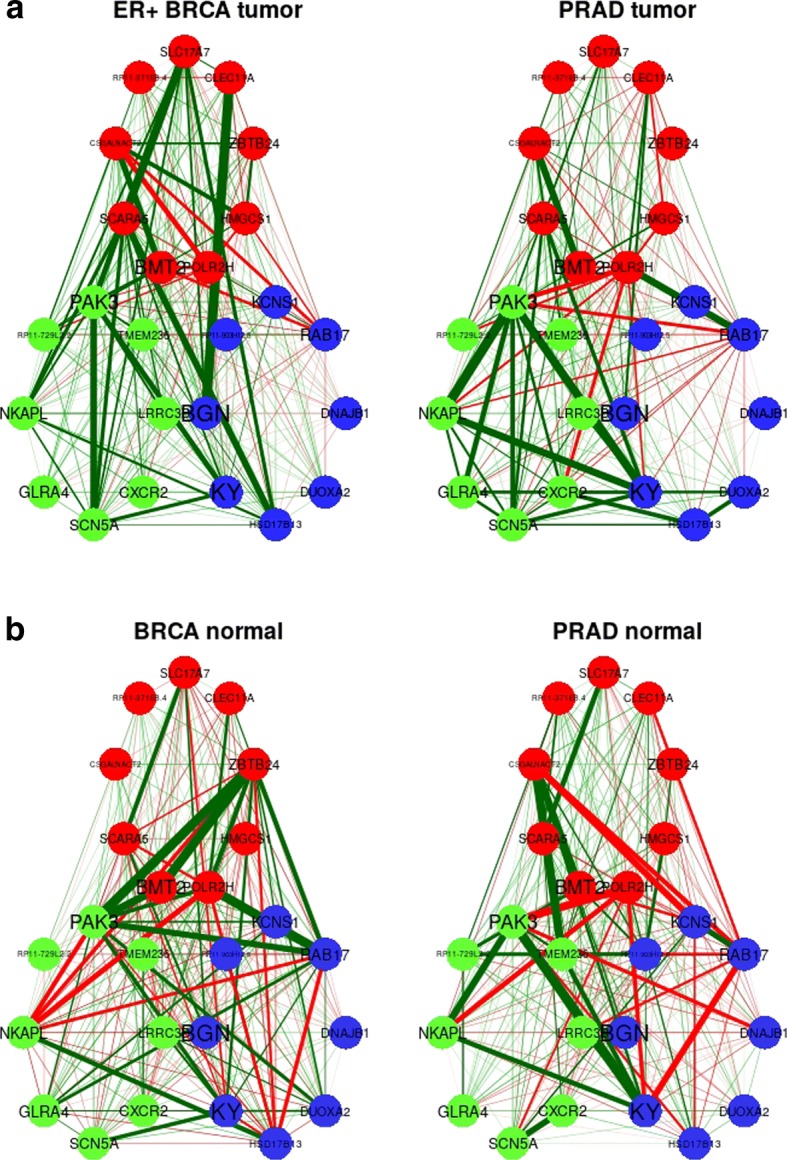
Network representation of the correlation between the genes selected by elastic net (EN) and twiner in the ER+ BRCA vs. PRAD case study; **a**) and **b**) stand for *tumor* and *normal* samples, respectively. ER+ BRCA, estrogen receptor positive Breast Invasive Carcinoma; PRAD, Prostate Adenocarcinoma

Figure 5 shows the correlation gene networks for the ER+ BRCA and PRAD *tumor* and *normal* samples regarding the genes exclusively selected in more than 75 BRCAPRAD EN and twiner models built on 100 randomly selected training and test sets, as well as the genes selected in common by the two model strategies. The thickness of the connecting lines represents the strength of the correlation, whereas green represents a positive correlation and red signals a negative correlation. In the search for shared disease biomarkers, and towards laboratory and clinical validation, particular attention might be given to the similarities between the relationships across less penalized selected genes (red coloured genes) and common (green) genes in ER+ BRCA and PRAD tumor samples, compared to that observed for BRCA and PRAD normal tissue samples.

#### AR+ TNBC vs. PRAD

With the goal of finding a common gene signature in AR+ TNBC and PRAD, EN and twiner were applied to the TNBCPRAD dataset. A less pronounced model accuracy improvement was obtained for twiner over EN, compared to the BRCAPRAD case, with a median MSE decrease in 5% for the training set (Table 2).

Thirteen genes were selected in more than 75 out of 100 twiner models (Fig. 3; Table 3), 5 in common with EN (*BGN*, *DNAJB1*, *GLRA4*, *GSTM3* and *NKAPL*), and the remaining 8 (*CD300LG*, *CTU1*, *KLHL4*, *PARK2*, *SCARA5*, *SLC35E2*, *SNCG* and *UCN*) exclusively selected by twiner (Fig. 3b; Table 3). A total of 5 genes were selected in common between twiner and the individual AR+ TNBC model (*BGN*, *CD300LG*, *NKAPL*, *PARK2* and *SCARA5*) and 4 (*CTU1*, *DNAJB1*, *KLHL4* and *NKAPL*) with the PRAD model (Table 3). From the 8 genes exclusively selected by twiner (Fig. 3b), particularly 2 (*SLC35E2* and *UCN*) have lower weights compared to the remaining genes exclusively selected by EN and selected in common by the two strategies (Table 3; Fig. 4b), corresponding to two genes that show a similar correlation pattern across AR+ TNBC and PRAD cancer types, and that are relevant in the classification of breast/prostate tissue into *tumor* (AR+ TNBC/PRAD) and *normal* tissue.

**Fig. 6:**
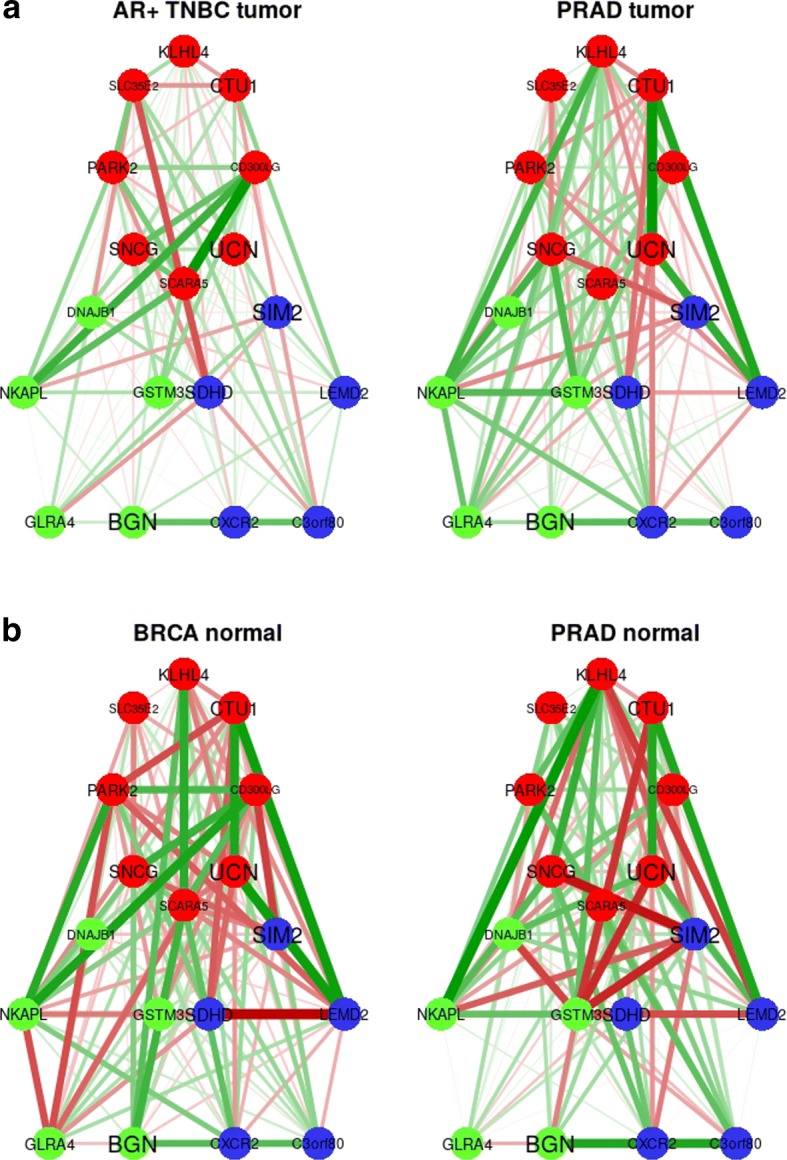
Network representation of the correlation between the genes selected by elastic net (EN) and twiner in the AR+ TNBC vs. PRAD case study; **a**) and **b**) stand for *tumor* and *normal* samples, respectively. AR+ TNBC, androgen receptor positive Triple-Negative Breast Cancer; PRAD, Prostate Adenocarcinoma

As for the ER+ BRCA vs. PRAD case, the correlation networks for the genes selected were obtained for the AR+ TNBC vs. PRAD *tumor* and *normal* samples (Fig. 6). In both case studies, the relationships highlighted in the correlation networks for the relevant genes selected by our analysis is expected to be matter for further disease understanding and biomarker research.

### Survival analysis

With the goal of assessing the clinical significance of accounting for the variables selected by our method, the genes selected by EN and twiner were tested in a survival analysis using the Cox regression model on ER+ BRCA, AR+ TNBC and PRAD tumor data. This constitutes an external and independent evaluation of the genes selected under the context of survival analysis, with the goal of expanding the usefulness of this approach to different clinical data types. Indeed, if the common gene signatures identified are also associated with the distribution of follow-up times and status, this would strengthen correlation-based regularization as a promising method to support prognostic assessment in cancer studies.

**Fig. 7:**
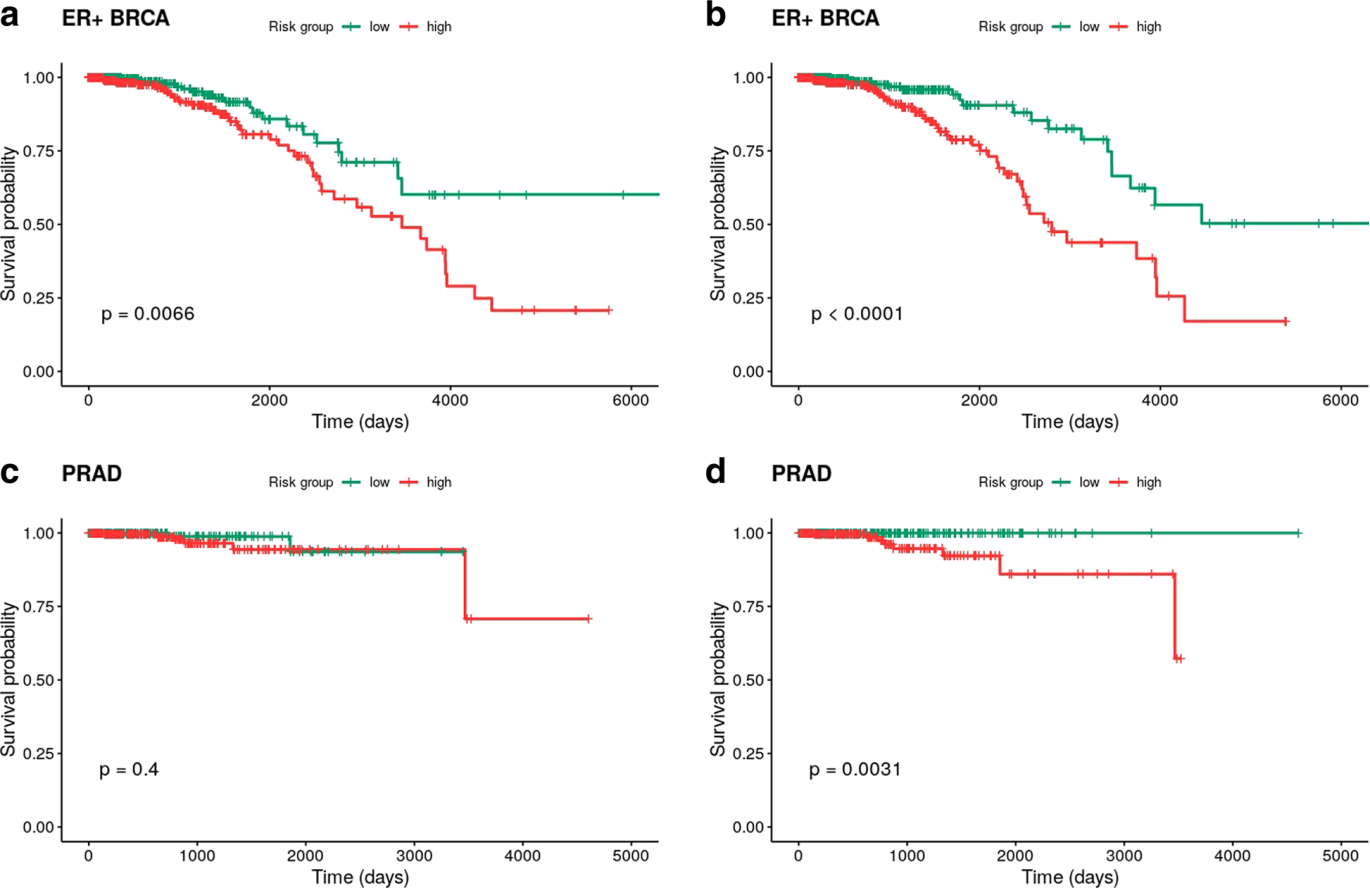
Kaplan-Meier survival curves obtained for the ER+ BRCA and PRAD datasets using the variables selected by elastic net (EN) (**a** and **c**) and twiner (**b** and **d**). ER+ BRCA, estrogen receptor positive Breast Invasive Carcinoma; PRAD, Prostate Adenocarcinoma

**Fig. 8:**
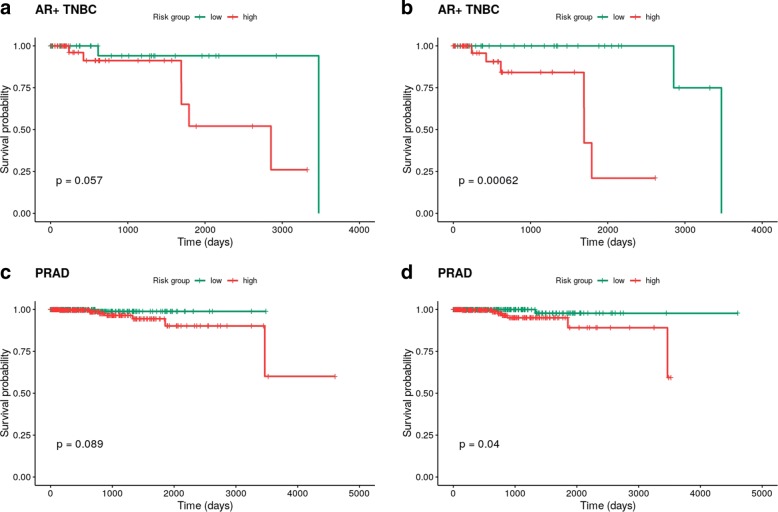
Kaplan-Meier survival curves obtained for the AR+ TNBC and PRAD datasets using the variables selected by elastic net (EN) (**a** and **c**) and twiner (**b** and **d**). AR+ TNBC, androgen receptor positive Triple-Negative Breast Cancer; PRAD, Prostate Adenocarcinoma

Figures 7 and 8 show the survival curves obtained for each dataset considered in the ER+ BRCA vs. PRAD and AR+ TNBC vs. PRAD cases, respectively. In the first case (Fig. 7), significance by the log-rank test for the difference in the survival distributions for high and low risk patients (separated by the median of the fitted relative risk) is highly increased in ER+ BRCA, while for PRAD the difference becomes significant. In the second case (Fig. 8), the difference for high and low risk individuals becomes statistically significant in both AR+ TNBC and PRAD. These results clearly indicate that accounting for genes showing a similar correlation pattern across the diseases, and without losing predictive ability, indeed improves the separation of high and low-risk patients.

## Discussion

After analyzing the computational results, an evaluation of the role of the genes selected in the diseases studied becomes crucial when the goal is to unravel new targets to tackle shared disease features. A discussion on the relevance of the selected genes on the clinical and oncobiology of breast and prostate cancers can be found next.

ER+ BRCA is the most frequent subtype of breast cancer amongst women. ER+ BRCA shares with PRAD the response to hormone signaling and marked bone osteotropism. Bone metastases are the principal site of metastasis in ER+ BRCA and PRAD, significantly affecting morbidity and mortality. However, it is still unknown if and how hormone signaling is involved in specific biological features of bone tropic metastasis initiating cells.

In this work we proposed to identify events specifically deregulated in both ER+ BRCA and PRAD cancers. We hypothesized that these events could reflect a possible association with bone tropism and/or hormone signaling. Our analysis led to the identification of nine genes exclusively selected by twiner, five identically deregulated in ER+ BRCA and PRAD (Table 3). Amongst these, three genes were up-regulated (*CLEC11A*, *POLR2H* and *ZBTB24*) and two down-regulated (*BMT2* and *SCARA5*). Amongst the three up-regulated, *CLEC11A* may be directly implicated in bone tropism or bone metastasis development.

*CLEC11A* was previously found to be part of a gene set up-regulated in cancer stem cell populations upon therapeutic insult [28]. *CLEC11A* encodes a C-type lectin domain protein, osteolectin, which is an osteogenesis driver usually secreted by bone stromal cells that promotes the differentiation of mesenchymal progenitors into mature osteoblasts [29]. Therefore, identification of *CLEC11A* in our model may be associated with a blastic bone metastasis phenotype, typical in PRAD and also frequent in BRCA.

*In silico* analysis had previously identified *POLR2H* as one of the key genes involved in the occurrence of PRAD, and *POLR2H* protein was significantly upregulated in PRAD tissues [30]. However, its role in cancer needs to be further explored.

Finally, *ZBTB24* encodes the poorly characterized zinc finger and BTB domain containing 24 protein, which belongs to the large ZBTB family of transcriptional repressors. Although its function is still unknown, it was recently shown that *ZBTB24* is involved in the control of DNA methylation [31]. It will be important to address its specific role in BRCA and PRAD.

Amongst the down-regulated genes, *SCARA5* (Scavenger receptor class A member 5) is a candidate tumor suppressor in several malignancies; however, its role in BRCA cell growth and metastasis is still unclear. *SCARA5* was found to be down-regulated in BRCA tissues and cells and correlated with clinicopathologic characteristics [32]. In this study, *SCARA5* overexpression significantly suppressed cell proliferation, colony formation, invasion, and migration, and induced G0/G1 arrest and apoptosis. Recently, another group reported that *SCARA5* expression was significantly decreased in tumors (92.2%), compared to non-cancerous tissue samples, due to the hypermethylation of the promoter [33]. There are no reports implicating *SCARA5* in PRAD, but based on our results we hypothesize a similar pattern of expression. *BMT2* has not been previously implicated in BRCA or PRAD.

AR (androgen receptor)-signaling is particularly important in prostate cancer, however, AR is also expressed in up to 90% of ER+ BRCA, and to a lesser degree, in *HER2* amplified tumors [34]. Although in BRCA HER2+ AR does not seem to play a role, in ER+ BRCA, AR signaling has been correlated with a better prognosis due to its inhibitory activity, but it also may increase resistance to anti-estrogen therapies such as tamoxifen. AR blockade can resensitize cells, and therefore is potential target in ER+ breast cancer. In TNBC, gene expression profiling studies have led to the identification of a luminal androgen receptor (LAR) subtype that is dependent on AR signaling, and there seems to be an association between AR expression and improved outcomes in TNBC. Clinical studies targeting AR have indeed shown promising results in this setting. Although to a significant less extent, TNBC may also metastize to the bone, however, the incidence of bone metastasis is significantly higher for the LAR subtype [35]. Therefore, we interrogated if we also could find common genes deregulated in TNBC with elevated AR expression, AR+ (AR values > median AR expression) and PRAD, AR-dependent. We found seven genes equally deregulated in both AR+ TNBC and PRAD (Table 3): *CTU* and *UCN*, up-regulated; and *CD300LG*, *KLHL4*, *PARK2*, *SCARA5* and *SNCG*, down-regulated. Only *SCARA5* was detected in the ER+ BRCA vs. PRAD analysis, suggesting specificity for AR+ TNBC.

*CTU1* (cytosolic tRNA thiouridilase) is involved in maintaining genome stability, since post-transcriptional modifications of transfer RNAs (tRNAs) at the wobble uridine 34 (U34) base are highly conserved and contribute to translation fidelity [36]. Partner enzymes in U34 tRNA modification, including ELP3 and CTU1/2 were found to be up-regulated in human breast cancers and sustain metastasis, through the translation of the oncoprotein DEK, that promotes the translation of the pro-invasive transcription factor LEF1 [37].

*UCN* (urocortin) has been previously reported to attenuate TGF *β*1-induced *Snail1* and *Slug* expressions, both in ER+ BRCA and TNBC in vitro models [38], suggesting that urocortin may inhibit TGF *β*1 oncogenic signaling and ultimately EMT. Therefore, *UCN* up-regulation would be associated with a better prognosis, a less invasive disease. However, it was also suggested that urocortin may have a dual role in cancer, since it differentially binds to CRFR1 or CRFR2 and either activates or blocks the Bcl-2/Bax/caspase-9 axis, leading to apoptosis or survival, respectively [39]. In clinical samples, *UCN* was found to be elevated in PRAD, although no correlations with clinical features were presented in this study [40].

The above described *SCARA5*, and also *SNCG*, *PARK2*, *CD300LG* and *KLHL4*, were down-regulated in both AR+ TNBC and PRAD.

The loss of epigenetic control of *SNCG* (synuclein-gamma) seems to be a molecular indicator of metastasis in a wide range of human cancers, including BRCA and PRAD [41]. In this case, is the reactivation of *SNCG* gene expression by DNA demethylation the contributing factor to malignant progression of many solid tumors and its expression in primary carcinomas is an effective molecular indicator of distant metastasis. Silencing *SNCG* in a prostate cancer cell line has shown to decrease proliferation and invasion in vitro, and tumor growth in vivo, with the exception of castrated mice [42], suggesting AR-dependence. It was shown that *SNCG* interacts with AR and promotes prostate cancer cellular growth and proliferation by activating AR transcription in an androgen-dependent manner, whereas *SNCG* was almost undetectable in benign or androgen-independent tissues prostate lesions. *SNCG* in PRAD was also described to be activated by Cav-1 in the tumor microenvironment [43]. Nevertheless, decreased expression of *SNCG* may correspond to a more indolent disease. In breast cancer, it was shown that TNBC cell lines do not express *SNCG*, in accordance with our results [44]; although in another study using a small cohort of 55 cases there was no association between the clinicopathologic parameters including histologic grade, ER positivity and HER2 status and the level of *SNCG* [45].

*PARK2* (PARKIN, E3 ubiquitin ligase) is involved in autosomal recessive parkinsonism. *PARK2* presents a partial mitochondrial localization at the outer mitochondrial membrane and its depletion results in abnormal mitochondrial morphology. An *in silico* analysis has shown that *PARK2* may be related to cell cycle control, suggesting a role in carcinogenic processes [46].

In accordance to our findings, *CD300LG* was found to be down-regulated in AR+ TNBC tissues when compared with adjacent normal studies [47], but its role in cancer is still unknown. This gene encodes the CD300 antigen-like family member G protein, also called nepmucin or CLM-9, expressed extensively in a variety of organisational venules and capillary endothelial cells in many organs. It is hypothesized that it may be involved in recruitment of immune cells, and that *CD300LG* down-regulation results in immune escape of cancer cells [48].

Also *KLHL4* (Kelch like family member 4) was expected to be down-regulated in breast cancer. A previous study has shown that this gene is down-regulated downstream of *IGFBP5*, silenced in response to stromal cells in ER *α*-positive breast cancer cells [49]. As this is part of a mechanism of induced resistance to anti-estrogen therapy, *KLHL4* down-regulation is expected to be associated with worse prognosis.

From the biological interpretation above, several links between the results obtained by our method and disease biology have been established, which reinforces the ability of our method to identify shared disease features in breast and prostate cancers. Moreover, these genes are able to stratify patients into high or low risk, according to overall survival, and deserve further studies to clearly determine their role in the progression of BRAC and PRAD.

## Conclusions

High-dimensional data leads to ill-posed inverse problems that cannot be tackled easily. Regularized optimization is a promising strategy to cope with undetermined problems, since it adds extra constraints to the loss function, which, if chosen carefully, can provide biological and clinical insight. We presented the twiner penalty, a correlation-based regularizer designed to enable the selection of similarly correlated genes in two diseases by sparse logistic regression, as a strategy to identify common key players in both diseases. The usefulness of the strategy proposed is shown in the context of Breast Invasive Carcinoma (BRCA) and Prostate Adenocarcinoma (PRAD), which show remarkable similarities at the hormone-related signaling pathways level. While being largely supported by the literature and clinical evidence by survival analysis, our results identified putative disease biomarkers which are expected to greatly improve our knowledge on the diseases and contribute to the definition of new target therapies.

## Data Availability

All the implementations described can be found in a R Markdown document available at https://github.com/sysbiomed/twiner, which allows full reproducibility and adaptation to new datasets.

## Abbreviations

AR: Androgen receptor
AUC: Area under the curve
BRCA: Breast invasive carcinoma
CDS: Coding sequence
EN: Elastic net
ER: Estrogen receptor
FPKM: Fragments per kilo base per million
HER2: Human epidermal growth factor receptor 2
MSE: Mean squared error
PC: Principal component
PCA: Principal component analysis
PR: Progesterone receptor
PRAD: Prostate adenocarcinoma
RNA-Seq: RNA sequencing
TCGA: The cancer genome atlas
TNBC: Triple-negative breast cancer
TWINER: Twin networks recovery

## Footnotes

1
https://github.com/averissimo/brca.data/releases/download/1.0/brca.data_1.0.tar.gz

2
https://github.com/averissimo/tcga.data/releases/download/2017.07.21-prad/prad.data_1.0.tar.gz
